# A Study of QT Interval and QT Dispersion During Laparoscopic Cholecystectomy

**Published:** 2009-04

**Authors:** Heena Parikh, Malini Mehta

**Affiliations:** 1 Assistant Professor, Department Of Anaesthesiology, Government Medical College, New Civil Hospital, Surat; 2 Professor And Head, Department Of Anaesthesiology, Government Medical College, New Civil Hospital, Surat

**Keywords:** Laparoscopic cholecystectomy, Electrocardiography, QT Interval, QT Dispersion

## Abstract

**Summary:**

The study was carried out randomly in 20 patients of ASA grade I and II of either sex, between the ages of 25 and 65 years scheduled for laparoscopic cholecystectomy. We studied the effect of intraperitoneal CO_2_ insufflation with head up position, on RR interval, QT interval, QTc interval, QT Dispersion, QTc Dispersion using computerized measurement with the help of 12 lead ECG.

The RR interval did not change significantly during the study but there was statistically significant increase in QT interval, QTD and QTcD.

## Introduction

Minimally invasive surgery has been the most significant development in general surgery as it results in tiny scars and early discharge from the hospital.

The introduction of laparoscopic cholecystectomy has seen it emerge as the gold standard for the procedure. Equally relevant, it remains the sole procedure performed by the vast majority of general surgeons. Recently much attention has focused on variations of ventricular repolarization because of their relation to cardiac arrhythmia due to prolonged CO_2_ insufflation with head up position in laparoscopic cholecystectomy.^1^,^2^

The purpose of this study was to determine the influence of a longer duration of CO_2_ insufflation on QT interval and QTD during laparoscopic cholecystectomy between the ages of 25 and 65 years.

QT Dispersion is defined as the difference between longest QT interval in any lead and the shortest, for a given set of electrocardiographic leads.^3^

## Methods

After obtaining the approval from hospital ethics committee, we studied of QT interval and QT dispersion during laparoscopic cholecystectomy randomly in 20 patients of ASA I & II, of either sex, between the ages of 25 and 65 years scheduled for laparoscopic cholecystectomy.

A detailed history was taken and all patients were examined thoroughly on previous day before surgery and informed consent was obtained. All patients with heart disease and lung disease were excluded from the study. In pre-operative room pulse, BP, SpO_2_ were monitored. No patients had received any medication. All patients were pre-medicated with IM glycopyrrolate (0.2 mg), IM tramadol (2 mg.kg^−1^), IV ranitidine (1 mg.kg^−1^) and IV ondansetron (0.08 mg.kg^−1^) half an hour before surgery.

After arriving at the operation room, standard 12 lead digital ECG (BPL CARDIART 8408 VIEW) was attached. Non-invasive blood pressure, SpO_2_, P_ET_CO_2_, respiratory rate, temperature (BPL MULTIPARA MONITOR MPM 5553 ACCURA) were monitored before anaesthetic induction (baseline), before CO_2_ insufflation, every 30 min for 150 min during CO_2_ insufflation, five minutes after deflation and at the end of surgery.

Anaesthesia was induced with propofol 2 mg.kg^−1^ and tracheal intubation was facilitated with succinylcholine 2 mg.kg^−1^. Anaesthesia was maintained with 66% nitrous oxide in O_2_ supplemented with vecuronium and isoflurane 1-2%. Ventilation was controlled and P_ET_ CO_2_ was maintained between 35 and 40 mm of Hg before CO_2_ insufflation. All patients received a crystalloid solution and continuous infusion of propofol 4 mg.kg^−1^.hr^−1^ during the study.

A veress needle was inserted at the umbilical level and connected to the CO_2_ insufflator, achieving and maintaining an intraabdominal pressure 10 to 12 mm of Hg. The minute ventilation was adjusted to maintain P_ET_ CO_2_ between 35 to 40 mm of Hg during the procedure. Patient was moved from supine to head up tilt of 20 degrees during the surgery.^4^,^5^ In all patients, the duration of intraperitoneal CO_2_ insufflation was between 120 to 150 minutes.

From each ECG, consecutive beat-to-beat data were digitally recorded and taken in print out form. QT intervals were determined by the use of newly developed software (BPL CARDIART 8408 VIEW) that detects the onset of Q wave and the end of T wave.^6^ QT intervals, QTc, QTD, QTcD were measured MAP, PR, RR interval, P_ET_CO_2_, SpO_2_, temperature were monitored continuously but for the purpose of study they were reached at the same interval. At the end of surgery, neuromuscular blockade was reversed with neostigmine 0.05 mg.kg^−1^ and glycopyrrolate 0.08 mg.kg^−1^.

An unpaired students t-test was applied for data analysis. Mean, standard deviation, variance and p value were taken with the help of EPI_6_ software. Probability values <0.05 were considered significant.

## Results

The mean age(years), sex ratio and mean weight(kg) of the patient were 52.35 ± 5.05, 8:12 (M:F) and 64.1 ± 4.17 respectively. Pulse rate, RR interval showed in Table–1 did not change significantly during the study period. Similarly SpO_2_ (98.39 ± 0.293) and temperature (98.4 ± 1.01) did not change significantly during the study period. MAP and P_ET_CO_2_ showed in Table–1 increased significantly during CO_2_ insufflation.

**Table – 1:** Changes in MAP, Pulse Rate, RR Interval and P_ET_CO_2_

	MAP		Pulse Rate		RR Interval		P_ET_CO_2_	
	Mean ±SD	p Value Vs baseline	Mean±SD	p Value Vs baseline	Mean±SD	p Value Vs baseline	Mean±SD	p Value Vs baseline
Baseline	99.69±2.53		81.4±7.65		741±72.63		39.95±87.47	
Before CO_2_	100.17±2.70	0.94	82.25±7.20	0.87	734±67.48	0.88	41.25±7.04	0.67
30 min.	100.12±3.17	0.94	82.7±8.08	0.81	731.8±75.11	0.85	40.2±6.76	0.93
60 min.	103.6±3.17	0.56	84.05±6.97	0.63	718.25±63.25	0.64	40.6±5.14	0.82
90 min.	105.3±3.32	0.005	85.45±7.84	0.47	708.5±73.46	0.51	45.1±6.76	0.10
120 min.	121.38±2.96	0.005	86.6±8.13	0.66	698.5±71.85	0.38	54.1±3.65	0.000097
150 min.	121.8±2.08	0.004	84.95±7.40	0.88	711.25±65.95	0.54	52.6±4.55	0.00035
5 min. after deflation	110.3±4.11	0.133	84.25±7.21	0.97	716±67.05	0.61	46.1±3.67	0.047
End of Surgery	105.19±3.03	0.42	83.25±7.39	0.87	725.5±68.57	0.74	42.3±4.92	0.43

MAP= Mean Arterial Pressure (mm Hg), Pulse rate = per minute, RR interval = msec, P_ET_CO_2_ = End tidal CO_2_ (mm Hg)

**Fig 1:**
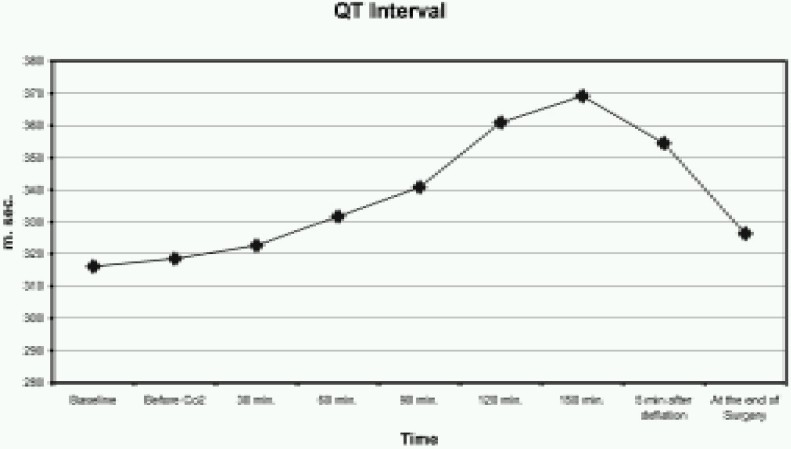
Changes in the QT interval before, during and after CO_2_ insufflation

**Fig 2:**
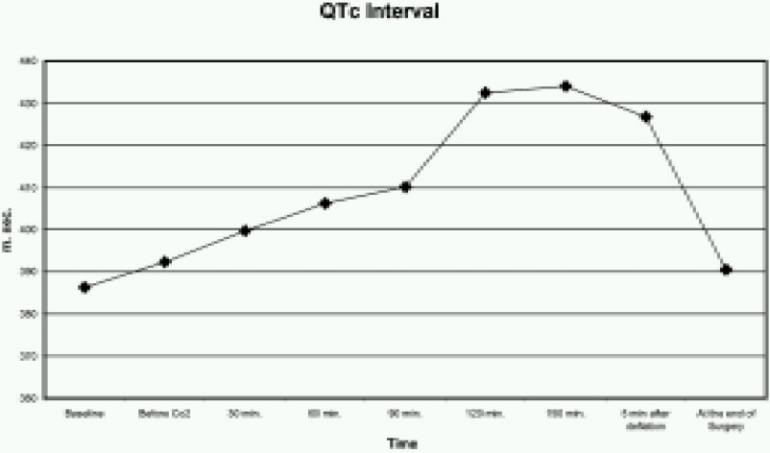
Changes in the rate corrected of QT interval (QT c) before, during and after CO_2_ insufflation

**Fig 3:**
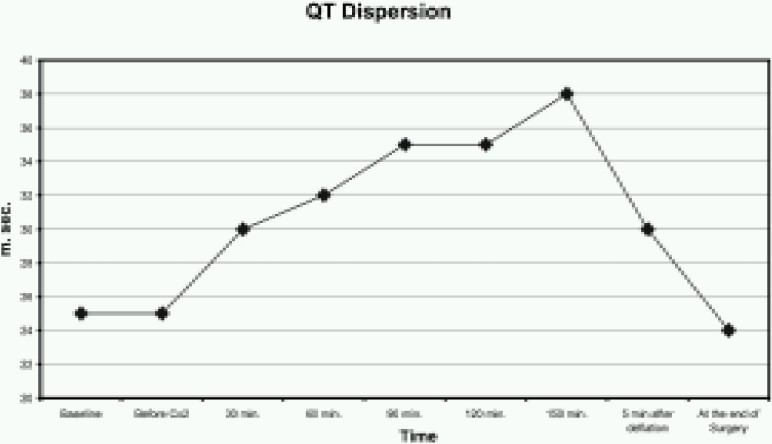
Changes in the QT dispersion (QTD) before, during and after CO_2_ insufflation

**Fig 4:**
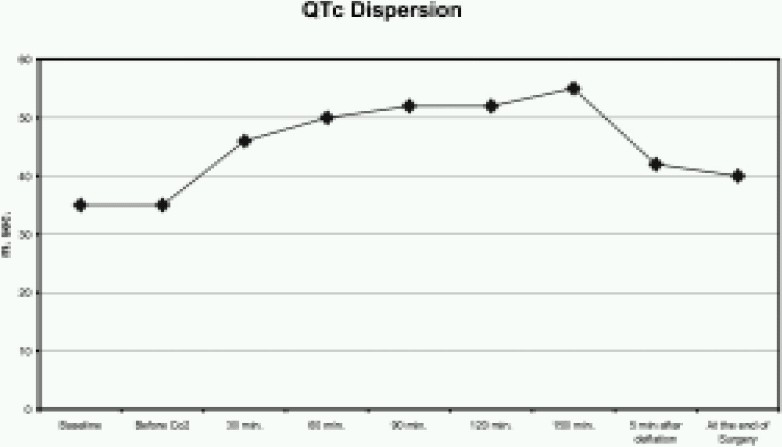
Changes in the rate corrected QT dispersion (QTcD) before, during and after CO_2_ insufflation

The QT interval was significantly greater from 120 to 150 minutes after the start of CO_2_ insufflation (*p* <0.005) (Fig 1). Similarly, QTc was also increased from 120 to 150 minutes after the start of CO_2_ insufflation (*p* <0.005) (Fig 2). The QTD and QTcD increased significantly from 120 to 150 minutes after the start of CO_2_ insufflation (*p* < 0.001) (Fig 3 & Fig 4).

## Discussion

We did not observe changes in PR but MAP was increased during intraperitoneal CO_2_ insufflation as similar to study by Joris, et al.^7^

Intraperitoneal CO_2_ insufflation during laparoscopic cholecystectomy induces hemodynamic changes.^4^ Administration of increasing concentrations of isoflurane via its vasodilator activity may have partially blunted these hemodynamic changes.^7^

QT interval, QTc interval, QTD and QTcD significantly increased after the start of CO_2_ insufflation from baseline. The reason is intraperitoneal CO_2_ insufflation, head up tilt, hypercapnia, inhaled anaesthetics and surgical stress may exert an influence on the QT interval and QTD.^5^,^8^

The effect of intraperitoneal CO_2_ insufflation on the QTD during laparoscopy is of considerable interest and the effect of a longer duration of CO_2_ insufflation on the QTD during laparoscopy remains unclear. Head up tilt induces prolongation of the QTD and QTcD due to increased sympathetic activity or decreased vagal tone.^1^ Hypercapnia produces an increase of QTc interval and QTD since laparoscopy cholecystectomy with CO_2_ insufflation may induce hypercapnia.^2^

PaCO_2_ should be maintained within normal limits by adjusting minute ventilation during the procedure.

In our study arrhythmia was not observed may be because we have not included cardiac disease patients.

We did not find any reference suggesting precautions that could be taken at what level but according to many studies^9^,^10^ arrhythmia is observed when QT and QTD increased two to two and half times than normal. Arrhythmia developed at any time irrespective of definite increase of QT and QTD^11^–^13^. So, precautions should be started at any level of increase from baseline. When QT & QTD is increased two to two and half times than normal, they considered at risk^9^,^10^. This increase is observed during 120 to 150 minutes of CO2 insufflation^5^. Pre operatively QT, QTD was assessed in every patients. QT and QTD increases intraoperatively even in ASA class I & II as observed in our study. So risk factors should be based on that.

It is concluded that QT dispersion is a simple, non-invasive measurement from a standard 12-lead digital ECG and it makes a significant contribution to identifying the patients at risk for life threatening arrhythmias.

As we studied small size population, still more study is required to reach upto definite conclusion.
